# Optimizing Therapeutic Strategies for Syringomyelia Associated with Tethered Cord Syndrome: A Comprehensive Review

**DOI:** 10.3390/children11080961

**Published:** 2024-08-09

**Authors:** Mohammad Mohsen Mosleh, Moon-Jun Sohn

**Affiliations:** 1 Department of Medicine, Graduate School of Medicine, Inje University, 75 Bokji-ro, Busanjin-gu, Busan 47392, Republic of Korea; mmohsenmosleh@oasis.inje.ac.kr; 2 Department of Neurosurgery, Neuroscience and Radiosurgery Hybrid Research Center, Inje University Ilsan Paik Hospital, College of Medicine, Juhwa-ro 170, Ilsanseo-gu, Goyang City 10380, Republic of Korea

**Keywords:** syringomyelia, dermal sinus tract, tethered cord syndrome, spinal dysraphism, surgery

## Abstract

This review highlights the need for therapeutic guidelines for syringomyelia associated with tethered cord syndrome (TCS) caused by spinal dysraphism (SD). A comprehensive literature review was conducted, selecting twelve articles to analyze common therapeutic strategies. Surgical cord untethering alone has recently become a preferred treatment, with 45 ± 21.1% of patients experiencing remission or improvement, 47 ± 20.4% unchanged and asymptomatic, and 4 ± 8% worsened. Untethering with direct surgical drainage for the syrinx had better outcomes than untethering alone (78% vs. 45%, *p* = 0.05). Terminal syringostomy was beneficial for syrinxes extending to the filum terminale but not for asymptomatic small syrinxes with a syrinx index < 0.4. Syrinx shunting was recommended for symptomatic large syrinxes (>2 cm in length and syrinx index > 0.5). Various shunt procedures for syrinxes are still advocated, mainly for refractory syringomyelia in Chiari malformation, posttraumatic cases, SD, or other causes. Personalized surgical methods that address the root cause of syringomyelia, particularly those improving cerebrospinal fluid flow, offer promising results with minimized complications. Ongoing studies are required to enhance management strategies for syringomyelia associated with TCS, optimize patient outcomes, and reduce the risk of recurrent symptoms.

## 1. Introduction

Syringomyelia, a disorder characterized by the development of a fluid-filled cavity or syrinx within the spinal cord tissue or in the central canal, presents a significant therapeutic challenge, particularly when associated with tethered cord syndrome (TCS) [1]. TCS is a clinical diagnosis seen in patients with a low-lying conus, which can result from various conditions such as lipomyelomeningocele (LMMC), diastematomyelia, a thickened or fatty filum, and intradural lipoma [2]. TCS has been classified based on its etiology, clinical presentation, severity, and associated conditions [3]. Based on the presence of spinal dysraphism, tethered cord patients have been categorized into three groups: those with occult TCS who have cutaneous stigmata; those with truly occult spinal dysraphism lacking cutaneous signs, who later developed symptomatic TCS in adulthood; and those with stable myelomeningocele following repair [4]. Approximately 12% of syringomyelia cases are linked with TCS, although the direct pathophysiological connection between these conditions remains elusive [5]. Unlike syringomyelia related to craniovertebral junction or other intraspinal pathologies, where a clear relationship between altered cerebrospinal fluid (CSF) dynamics and syrinx formation is established, the mechanisms driving syringomyelia in TCS are poorly understood [6]. The clinical features caused by a tethered cord may significantly overlap with those originating from the syrinx, making a clear distinction challenging [7]. Nevertheless, a higher incidence of bladder, bowel, and gait problems, instead of motor and sensory deficits, have been reported in the TCS cases associated with a terminal syrinx compared to the TCS cases without a syrinx [7]. Once syringomyelia has developed, the elevated intramedullary pressure and the intra-syrinx fluid movements may lead to significant spinal cord injury [8]. Syringomyelia patients may develop various neurological deficiencies from untreated syringomyelia, some of which may persist despite surgical intervention [7]. Early detection, regular monitoring, and prompt treatment of the underlying cause are crucial for minimizing potentially irreversible neurological defects [7]. 

Modern diagnostic imaging technologies enable the detection of syringomyelia and its associated anomalies [9]. Dynamic magnetic resonance imaging (MRI) or cardiac-gated cine MRI flow studies provide a detailed, noninvasive assessment of cerebrospinal fluid (CSF) dynamics [9]. These techniques identify flow abnormalities at the foramen magnum, visualize spinal cord motion, and track syrinx fluid movement during cardiac cycles. Additionally, they are useful for documenting postoperative changes in CSF flow and measuring improvements [9]. Although the size, length, and number of syringomyelia are recognized as predictors of prognosis, a decrease in syrinx size is associated with a greater likelihood of motor improvement than sensory disturbance and pain [10]. Motor dysfunction, brainstem herniation, and basilar invagination are predictors of poor clinical prognosis [11]. Since syringomyelia is typically diagnosed concurrently with or as a consequence of its underlying cause, treatment decisions should aim to address the fundamental mechanisms leading to syringomyelia, with the primary goal of restoring normal CSF flow at the affected site [12]. Tethered cord surgery is indicated when the symptoms of tethered cord syndrome are significant and persist despite non-surgical treatments, when there are associated complex spinal pathologies, or when complications from previous interventions necessitate further surgical correction [13].

General treatment methods for syringomyelia vary according to the etiology and pathogenesis of the disease, ranging from simple approaches such as managing associated supratentorial hydrocephalus to more complex procedures like decompressing hindbrain abnormalities, resecting spinal arachnoiditis, or directly decompressing the syrinx [5]. In cases of Chiari I malformation, syringomyelia often results directly from impaired CSF flow at the foramen magnum [6]. Consequently, surgical intervention such as a suboccipital approach to foramen magnum decompression, which improves CSF flow, is a well-established treatment method to reduce syrinx size and alleviate symptoms [6]. Conversely, establishing treatment strategies and surgical plans for syringomyelia caused by TCS requires a complex evaluation due to the unclear relationship between tethered cord release and syrinx morphology [7]. There are still various opinions and controversies regarding the treatment of syringomyelia associated with TCS, necessitating further research and long-term follow-up to better understand this pathology and its management [2,14,15,16,17,18,19,20,21,22,23,24]. Despite advancements in neurosurgical techniques and a growing understanding of the pathophysiology of these conditions, there is considerable variability in reported outcomes and surgical strategies [2,14,15,16,17,18,19,20,21,22,23,24]. This variability underscores the need for a comprehensive literature review to evaluate current evidence, identify best practices, and highlight areas where further research is needed [2,14,15,16,17,18,19,20,21,22,23,24].

### Pathophysiology of Syrinxes Associated with Tethered Cord Syndrome

Syringomyelia is primarily understood through several hypotheses regarding its pathophysiology, with the most common being the obstruction of CSF flow, formation of pressure gradients, and a one-way valve mechanism [9,25,26,27]. In TCS, the abnormal tethering of the spinal cord can lead to localized compression at specific sites along the cord. This compression can disrupt the flow of CSF around the spinal cord, creating asymmetric resistance to CSF flow and resulting in pressure gradients between the central canal and the surrounding subarachnoid space, which can lead to CSF accumulation and the formation of a syrinx [1,9]. Additionally, this tethering may also act as a one-way valve, selectively allowing CSF to flow in one direction and thereby obstructing normal circulation and increasing internal pressure within the syrinx, contributing to syringomyelia [27]. Moreover, the movement of the tethered cord can resemble a piston-like motion, similar to that seen in Chiari malformation, enhancing direction-selective resistance to CSF flow and further promoting syrinx formation [27]. Altered CSF pulse pressure dynamics and ischemic factors are the hypotheses that best explain the development of syringomyelia associated with tethered cord syndrome [28,29]. TCS and arachnoiditis elevate flow resistance in the subarachnoid space, which is associated with a slight increase in the magnitude and/or a shift in the timing of the CSF pressure drop across the affected arachnoiditis region in relation to the cardiac cycle. This restriction alters the pressure dynamics of the CSF, causing abnormal pulsatile forces that can lead to the formation and expansion of a syrinx [29]. This phenomenon may arise in patients with adhesions from prior lipoma surgery or myelomeningocele (MMC) closure [7]. Additionally, tethering results in direct mechanical stretching and traction of the spinal cord during spinal flexion, causing the cord to temporarily expand with each instance of spine flexion [27]. Tensile radial stress may induce a syrinx in the previously normal cord tissue and transiently lower pressure can draw in interstitial fluid, causing the syrinx to expand if fluid exit is restricted [28]. This phenomenon may arise due to a stretched spinal cord caused by a thickened filum terminale. Additionally, the presence of a syrinx can create a feedback loop where the expanding cavity causes additional traction on the spinal cord, worsening the ischemic conditions. This can result in a range of symptoms, including pain, weakness, and sensory disturbances, depending on the location and extent of the syrinx [7]. Research by Yamada and colleagues suggests that the degree of caudal traction reduces blood flow and oxidative metabolism, causing cellular hypoxia and subsequent ischemic injury. This disruption in oxidative metabolism alters the redox ratio by reducing cytochrome a, cytochrome a3, and adenosine triphosphate, ultimately impairing nerve function. The ischemia-induced functional changes affect lower motor neurons, leading to dysfunction and symptoms in the lower extremities [30,31]. Surgical untethering can lead to varying levels of neurological improvement by restoring metabolic function and enhancing oxygenation. The extent of recovery largely depends on the severity and duration of spinal cord traction. Consequently, patients with mild to moderate traction generally experience full recovery, while those with severe traction often see only minimal or partial improvement [30,32]. This understanding underscores the importance of addressing both mechanical stress and fluid dynamics in the management of syringomyelia associated with TCS. Effective treatment strategies must consider these pathophysiological mechanisms to optimize patient outcomes and prevent the progression of syrinx formation.

This review examines therapeutic strategies for syringomyelia associated with TCS. The existing literature on treatment outcomes for syringomyelia in this specific condition is limited, and there is a lack of clearly established and standardized surgical protocols tailored to the diverse pathologies of TCS. Therefore, through this comprehensive analysis, we aim to evaluate the strengths and limitations of evidence-based surgical strategies for patients with these complex and interrelated conditions and to assist in the development of appropriate treatment strategies for this rare disorder.

## 2. Materials and Methods

**Figure 1:**
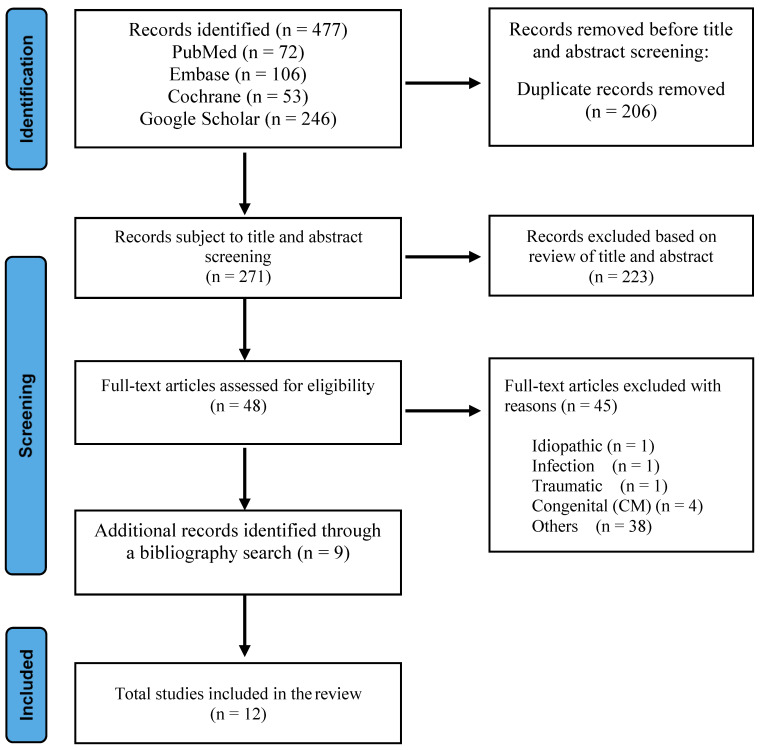
Flowchart of the process used to identify and screen studies for inclusion.

A literature search of Medline/PubMed, Google Scholar, Cochrane, and EMBASE databases was conducted, focusing on various surgical approaches for distal syringomyelia associated with tethered cord syndrome. Keywords and Medical Subject Headings terms used were consistent through all search engines and included “syringomyelia”, “spinal dysraphism”, “dermal sinus tract”, “tethered cord syndrome”, “surgical management”, “outcome”, and “complication”. English language literature published between July 2013 and July 2023 was gathered. The search encompassed original studies, meta-analyses, and randomized controlled trials. To complement the database search, we manually reviewed reference lists from articles deemed highly relevant to this topic. This step aimed to uncover seminal works and additional studies that might have been missed during the electronic search. The final outputs from the different databases were exported to and combined in EndNote 20, where duplicates were manually removed. We excluded articles unrelated to the interventions or outcomes of interest, including syringomyelia related to idiopathic causes, infection, inflammation, trauma, neoplastic conditions, congenital conditions, and untreated hydrocephalus to focus on the effects of untethering in cases where the underlying pathology is more amenable to surgical correction. During the initial screening process, 223 records were excluded based on the review of titles and abstracts, as these articles did not focus on syringomyelia associated with TCS, were primarily reviews, commentaries, or case reports lacking primary data, and many were centered on other diseases or conditions irrelevant to our review’s inclusion criteria. The final articles included in our study comprised 12 retrospective studies: four articles on surgical cord untethering alone, four articles on untethering with syrinx drainage, and four articles on syrinx shunting. Figure 1 shows the flow of information through the different stages of the review process.

We conducted a comprehensive analysis of data from 12 articles, which included studies on three primary therapeutic modalities for TCS-related syringomyelia: untethering alone, direct drainage including a terminal syringotomy, and shunt procedures. Each modality was evaluated using data from four articles. The data collection focused on the following variables: demographic features, clinical presentation, neurological status, preoperative and postoperative radiographic results, and overall outcomes according to the categorization of therapeutic modalities. The results of therapeutic modalities were summarized and categorized into surgical cord untethering alone, direct drainage including a terminal syringotomy, and syrinx shunt procedures. Postoperative outcomes were categorized into complete resolution, improvement, unchanged if no significant change in syrinx size, and worsened. Statistical analysis was performed on postoperative outcomes, (*p* < 0.05). A one-way ANOVA test was performed using GraphPad Prism 10.2.3 for Windows (GraphPad Software, Boston, MA, USA).

## 3. Results

The clinical features, outcomes, and summaries of the three treatment modalities are comprehensively analyzed and presented in tables. This structured approach allows for the identification and analysis of key factors influencing the treatment outcomes of syringomyelia induced by TCS, comparing the indications and results of each treatment method.

### 3.1. Surgical Cord Untethering Alone

TCS, a complex condition often associated with syringomyelia, is primarily treated with surgical untethering to alleviate symptoms and reduce syrinx size. Surgical untethering has recently emerged as a preferred treatment strategy for syringomyelia caused by TCS. Table 1 presents the clinical and radiological changes and prognostic outcomes from four retrospective studies focusing on untethering.

**Figure 2:**
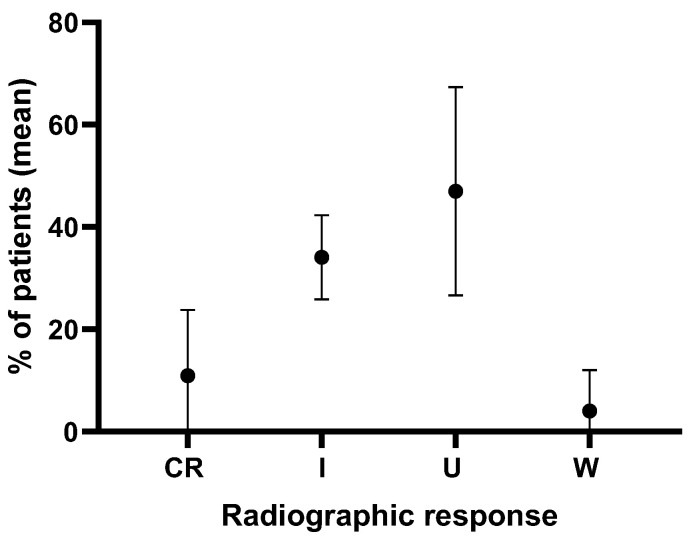
Analysis of radiographic syrinx outcomes in 128 cases over an average follow-up of 3.6 years in four retrospective studies on patients that underwent surgical cord untethering alone. CR = complete resolution, I = improvement, U = unchanged, W = worsened.

In four retrospective studies involving 128 patients, the postoperative outcomes of the syrinx were analyzed over an average follow-up period of 3.6 years. Radiographic results indicated that although significant improvement in syrinx index occurred in a wide range of variations, approximately 25% to 63% or 87.5%, 12.5% or 37% up to 75% showed no change, yet satisfactory clinical outcomes were reported. Statistically significant differences were observed between preoperative and postoperative syrinx measurements (*p* < 0.05) [23], despite the lack of a radiographic change in syrinx size ranging between 28% and 75% in the literature [2,20,21,23]. Averaging all results showed that 45 ± 21.1%, 47 ± 20.4%, and 4.0 ± 8% of patients achieved complete resolution/improvement, no change, and worsened syrinx indices, respectively (Figure 2) [2,20,21,23]. Despite the variability in results, significant clinical improvement is frequently observed postoperatively. However, changes in syrinx size do not consistently correlate with clinical outcomes, emphasizing the need for careful postoperative monitoring.

Therefore, the analysis of these retrospective data on surgical treatment shows that improvements in syrinx size have yielded satisfactory results. Notably, patients with no change in syrinx size also reported being mostly asymptomatic with favorable overall outcomes. However, when the syrinx worsens, it emphasizes the need for careful clinical and radiological follow-up. Additionally, the necessity for further studies on the long-term prognosis of large-sized syrinxes that remain unchanged is highlighted.

### 3.2. Direct Syrinx Drainage and Cord Untethering

Retrospective studies, as shown in Table 2, have demonstrated that combining direct syrinx drainage with untethering resulted in significantly better clinical and syrinx improvement outcomes compared to untethering alone (78% vs. 45%, *p* = 0.05) [18]. Specifically, research on terminal ventriculostomy for terminal syringomyelia reported 100% resolution of the syrinx (*p* < 0.005) and a 91% improvement in motor and sensory neurological deficits. 

Consequently, terminal ventriculostomy is particularly effective for syringomyelia extending to the terminal region of the conus. However, its efficacy is limited in asymptomatic cases or cases with a small syrinx with an index of less than 0.4 [24]. Among the three comparative studies between untethering alone (Group 1) and the combined direct drainage procedure (Group 2), two of these studies indicated that the rate of complete syrinx resolution and improvement in Group 2 was markedly higher (~80%) than those in Group 1 (26–42%). Conversely, the rate of unchanged syrinx size was markedly higher in Group 1 (56.2–73%) compared with Group 2 (21.4–30.7%) [17,18]. 

**Figure 3:**
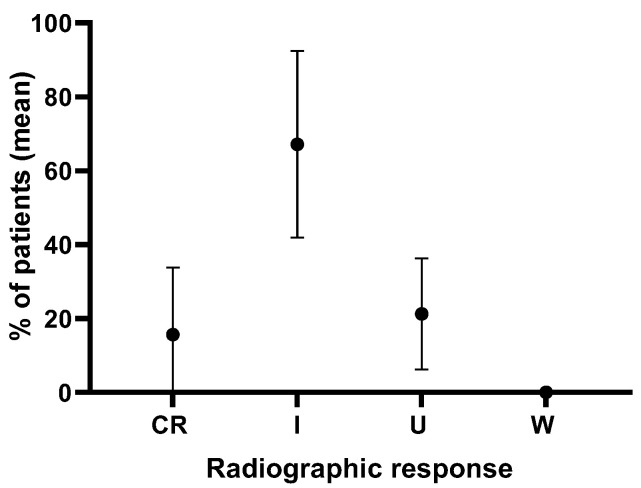
Analysis of radiographic syrinx outcomes in 89 cases in four retrospective studies over an average follow-up of 1.9 years from patients that underwent syrinx drainage combined with surgical cord untethering. CR = complete resolution, I = improvement, U = unchanged, W = worsened.

An analysis of 89 cases from four retrospective studies, with an average follow-up period of 1.9 years, where direct syrinx drainage and surgical cord untethering were performed, showed that the syrinx index was completely resolved and improved in 67.1 ± 25.3% of cases but remained unchanged in 21.2 ± 15.0% of cases (Figure 3) [17,18,19,24]. The combination of direct syrinx drainage with surgical cord untethering shows superior clinical and radiographic outcomes compared to untethering alone. Direct syrinx drainage, especially in cases with extensive syringomyelia, significantly enhances syrinx resolution and improves neurological function. The combined approach addresses the limitations of untethering alone by providing a more effective reduction in syrinx size and better clinical outcomes. However, its use should be carefully considered in cases with smaller syrinx, as the risk–benefit ratio may not be favorable.

In summary, combining surgical cord untethering with direct syrinx drainage demonstrates superior clinical and radiographic outcomes compared to untethering alone. This strategy offers a higher likelihood of syrinx resolution and clinical improvement, providing a more effective treatment option for patients with TCS and associated syringomyelia. Careful patient selection and meticulous postoperative monitoring are crucial, particularly in cases involving smaller syrinxes, to ensure optimal outcomes and manage potential risks effectively.

### 3.3. Syrinx Shunting

The pathophysiology of each syringomyelia is crucial in the determining of the treatment strategy, as shown in Table 3. Syrinx shunting has been safe and effective for idiopathic syringomyelia or cases with unknown causes, or when first-line treatments have been insufficient or infeasible. Improvement in clinical status often correlates with a reduction in the size of the syrinx. The radiographic picture of syringomyelia is an important indicator of clinical severity, and progressive scoliosis is a key indicator of the deterioration of syringomyelia. Table 3 demonstrates the surgical treatment of syringomyelia generally yields very good outcomes. Large symptomatic syrinxes (syrinx index ≥ 0.7) associated with open SD should be surgically treated. A syringo-subarachnoid shunt is effective in both closed and open SD-associated syringomyelia. Foramen magnum decompression is indicated for the treatment of syringomyelia with MMC. Large syrinxes with symptoms of MMC have been successfully treated by a syringo-subarachnoid shunt. Shunting is necessary and effective for syringomyelia with a large cyst, especially in the setting of progressive symptoms. TCS is an important condition to consider in patients with open SD and should be surgically treated when clinically or radiographically significant. Syrinx shunting can yield good radiographic and clinical outcomes during short-term follow-up. However, this initial improvement in syrinx outcomes diminishes markedly over an extended follow-up period. At three months of MRI follow-up, syrinx outcomes in terms of improvement, unchanged, and worsening were 90.2%, 7.3%, and 2.4%, respectively. These values dropped to 56%, 39.2%, and 4.8%, respectively, when the MRI follow-up duration was extended (31 ± 28 months) [22] (Table 3).

While syrinx shunting can provide short-term relief, its effectiveness diminishes over time, especially if the underlying cause of syrinx formation is not addressed. Surgical cord untethering, although less invasive, may not be sufficient for larger syrinxes, necessitating additional procedures such as a direct syringotomy/myelotomy or terminal ventriculostomy. The evolution of surgical techniques has relegated shunting to a secondary role, highlighting the importance of addressing the primary pathology to ensure long-term success and prevent neurological deterioration.

In summary, surgical cord untethering is less invasive and targets the underlying tethering that contributes to syrinx formation. Studies indicate significant improvements in many cases; although, some variability in clinical and radiographic results exists. Larger syrinxes, those with a syrinx index greater than 0.5–0.6, are less likely to resolve with untethering alone and may require additional procedures to directly address the syrinx. Combined procedures, such as a concomitant direct syringotomy/myelotomy or terminal ventriculostomy and spinal cord untethering, have shown better resolution rates and clinical improvements compared to untethering alone. Historically, in the 1990s, syrinx-shunting approaches including syringo-subarachnoid, syringo-pleural, and syringo-peritoneal shunts were employed earlier in treatment, but improvements in surgical techniques for spinal cord tethering have since made shunting a secondary option. Long-term follow-up shows a 50% failure rate over 10 years if the primary cause is not addressed. Therefore, shunt operations for syringomyelia are considered a last resort when other measures fail and syringomyelia threatens neurological function.

## 4. Discussion

### 4.1. Therapeutic Approaches for Syrinx Associated with Tethered Cord Syndrome

#### 4.1.1. Surgical Cord Untethering Alone

The initial therapeutic approach for syringomyelia associated with TCS often involves the observation of the syrinx following surgical untethering of the spinal cord. The rationale is that untethering can alleviate mechanical stress and improve CSF dynamics, potentially resolving the syrinx. The outcomes of surgical cord untethering alone for syrinx management are notably controversial, with a range of clinical and radiographic results reported. Statistically significant decreases in syrinx size postoperatively have been documented in some studies, indicating potential effectiveness (*p* < 0.05). However, the variability in radiographic outcomes is substantial, with 28% to 75% of cases showing no radiographic change in syrinx size post-surgery.

A detailed analysis of 128 cases from four retrospective studies over an average follow-up period of 3.6 years showed that syrinx resolution or improvement occurred in approximately 45% of cases, while 47% of cases showed no change, and 4% experienced a worsening of the syrinx index. This wide range of outcomes highlights the unpredictable nature of surgical cord untethering alone and suggests the need for more consistent and possibly combined treatment approaches. In a study of TCS patients, Rakip, et al. divided the patients into Group I (pediatric, n = 34) and Group II (adult, n = 20), and investigated the clinical and radiological characteristics of syringomyelia and other anomalies in pediatric and adult patients with TCS. They performed untethering surgery for all and syrinx drainage only for one patient. The syrinx outcomes in Group I and Group II were reported in terms of improved (44.4% vs. 50%), complete resolution (18.6% vs. 37.5%), and unchanged (37.5% vs. 12.5%), in each group respectively. When comparing preoperative versus postoperative improvement of the syrinx index in cases where the SI had decreased, they reported SI changes of (0.21 ± 0.15–0.14 ± 0.15) in Group I and (0.12 ± 0.17–0.05 ± 0.04) in Group II, with a significant *p*-value for both groups (*p* < 0.05). However, of these patients, 37% of the 27 patients with syringomyelia in Group I and 12.5% of 8 patients in Group II showed no change in the syrinx size. The adult group experienced fewer and milder symptoms and showed better surgical outcomes than the pediatric group [23]. 

In a study of 25 cases of syrinxes associated with TCS who underwent untethering surgery, Bruzek, et al. reported no significant postoperative changes in the length and width of syrinx; the average syrinx length increased by 0.86 ± 4.36 vertebral levels and width decreased by 0.72 ± 2.94 mm. Forty-eight percent of these cases showed no change in postoperative syrinx size [2]. Similarly, Kulwin, et al. reported no change in postoperative syrinx size in 77% of 16 cases of TCS with syringomyelia who underwent untethering of the cord. The preoperative average syrinx diameter (3.7 mm) indicated a trivial change (3.6 mm) postoperatively [21]. On the other hand, the study conducted by Lee, et al. on 33 cases of TCS with syringomyelia shows that untethering alone, in TCS with syringomyelia associated with OSD, is an effective management strategy and has favorable postoperative clinical and radiological outcomes. However, 28% of these cases did not show any changes in the size of syrinx during postoperative follow-up [20]. 

In summary, the analysis of postop syrinx outcomes in 128 cases over an average follow-up of 3.6 years in four retrospective studies that underwent surgical cord untethering alone indicated complete resolution/improvement, unchanged, and worsened syrinx index in 45 ± 21.1%, 47 ± 20.4%, and 4.0 ± 8% of the cases respectively [2,20,21,23]. While the relationship between a spinal cord syrinx and its primary pathology (e.g., Chiari-associated syrinxes) is well understood, the response of spinal cord syrinxes to surgical intervention for a tethered cord remains unclear [2]. Some authors have reported a lack of or insignificant correlation between clinical improvement and the radiographic resolution of the syrinx following interventions for tethered cord release [2,21]. Bruzek et al. have reported the long-term outcomes for patients with syrinxes who underwent either primary or secondary untethering operations for closed SD. In their cohort of 25 patients with syrinxes who underwent cord untethering, the authors concluded that in patients undergoing either primary or secondary untethering procedures for closed SD, the size of the syrinx did not consistently change, nor was there a correlation between changes in syrinx size and clinical improvement. The authors suggest that for patients with a tethered cord and syrinx, the decision to operate should prioritize symptoms related to the tethered cord and the primary SD rather than the presence of the syrinx, as they saw no clear evidence of syrinx size improvement following surgery [2]. Similarly, in a report on 16 patients with clinical symptoms of TCS and radiologic evidence of syrinxes which showed clinical improvement in all cases, only 25% indicated radiographic improvement in the syrinx, and only 2 (12.5%) had resolution of the syrinx. However, limited evidence has been reported on syrinx resolution after surgical cord untethering [21].

#### 4.1.2. Direct Drainage of the Syrinx Combined with Surgical Cord Untethering

The direct drainage procedure involves creating an opening in the syrinx to facilitate the outflow of the accumulated CSF, thereby relieving pressure on the spinal cord and surrounding tissues. This method can provide rapid symptomatic relief and significant improvement in syrinx size. Particular factors, such as the consistency of the syrinx with the central canal, the size of the syrinx, and the persistence of drainage, may further improve the outcomes of this method. Combining syrinx drainage with surgical cord untethering has shown more promising results compared to untethering alone. Outcomes from this combined approach demonstrate a significantly higher rate of syrinx resolution or improvement (78%) compared to untethering alone (45%, *p* = 0.05). Terminal ventriculostomy, a specific method of combined treatment, resulted in 100% syrinx resolution and 91% improvement in motor and sensory deficits for terminal syringomyelia cases (*p* < 0.005). This approach is particularly beneficial for syrinxes extending to the filum terminale but poses risks for smaller syrinxes (syrinx index < 0.4). Two additional studies further corroborated the superior outcomes of combined treatments, reporting that approximately 80% of patients experienced syrinx resolution or improvement compared to 26–42% in the surgical cord untethering alone group. Furthermore, the rate of unchanged syrinx size was significantly higher in the untethering alone group (56.2–73%) compared to the combined treatment group (21.4–30.7%).

An analysis of 89 cases from four retrospective studies over an average follow-up of 1.9 years found that combined syrinx drainage and surgical cord untethering led to syrinx resolution or improvement in 67.1% of cases, while 21.2% had unchanged syrinx indices. These data support the efficacy of combined surgical approaches for better syrinx management outcomes. Treatment of syringomyelia associated with TCS, whether through untethering alone or untethering combined with syrinx drainage, has been discussed in the literature. Erkan et al. divided patients into two groups based on the surgical approach: (1) those who had the tethered cord release procedure (Group 1, n = 16), and (2) those who had this procedure combined with syrinx drainage (Group 2, n = 14). After a year of follow-up, the post-operative syrinx index of Group 2 (0.18 ± 0.1) compared with that of the preoperative syrinx index of 0.45 ± 0.16 of Group 2 demonstrated a significant decrease (*p* = 0.001), which shows a significant change when compared with the same values in Group 1 (*p* = 0.13). Comparably, treatment in Group 2 resulted in better outcomes in terms of resolution of sensory deficits (*p* = 0.04) and urinary symptoms (*p* = 0.05). However, terminal ventriculostomy did not show satisfactory outcomes in which only 5/8 of procedures reduced syrinx size, probably due to early healing of the ventriculostomy site. Additionally, dorsal midline myelotomy, when performed in small syringes (SI < 0.4), resulted in additional neurological morbidities [18].

In a 3.5-year follow-up study of 34 patients, Beaumont et al. divided the patients into two groups: (1) the TCS group (n = 24) and (2) the TCS + TS group (n = 10). The incidence of TS was 29%; only one patient underwent surgical syrinx drainage, while the rest only had tethered cord release. All preoperatively asymptomatic patients remained asymptomatic postoperatively. In the TCS + TS group, all patients showed clinical improvement or became asymptomatic after the tethered cord release. In the TCS group without TS, most patients improved or became asymptomatic, although a few experienced no change or worsening symptoms. Patients without a preoperative syrinx did not develop one postoperatively. Postoperative MRI in a limited number of patients showed either no change or a reduction in syrinx size [19].

#### 4.1.3. Syrinx Shunting

In the 1990s, syrinx shunting was commonly used early in the treatment of syringomyelia. It was believed that shunting would be more successful if the syrinx was drained to a low-pressure area outside the theca, such as the peritoneal or pleural cavity, rather than the subarachnoid space [7,25]. The optimal method for syrinx shunting in patients with symptomatic syringomyelia remains a subject of debate [33]. A recent metanalysis indicates clinical improvement rates of 61% for syringo-subarachnoid shunts, 64% for syringo-peritoneal shunts, and 71% for syringo-pleural shunts [33]. A considerable number of patients may experience clinical deterioration after a syrinx shunt procedure, which is caused by spinal cord injury from the myelotomy [34]. The estimated rates of clinical deterioration after shunt placement are reported to be 13% for both syringo-subarachnoid and syringo-peritoneal shunts, and 10% for syringo-pleural shunts [33]. While all three types of shunts yield similar outcomes regarding clinical improvement and deterioration, syringo-peritoneal shunts have a higher incidence of malfunction requiring surgical revision. The syringo-pleural shunt, popular due to the anatomical proximity of the pleural space to the cervicothoracic syrinx and the negative pressure of the pleural space [35], may offer the highest rates of clinical improvement with the lowest need for reoperation [33]. Certain recommendations have been proposed to avoid syringo-pleural complications. Intraoperative neurophysiological monitoring using motor evoked potentials and somatosensory evoked potentials (SSEPs) is crucial during a myelotomy. If SSEPs are lost, the procedure should be paused to allow for recovery. The pleural entry site should be positioned below the scapula tip, at the level of the seventh thoracic vertebra, to reduce the risk of catheter dislodgment [36]. However, advancements in surgical techniques for spinal cord tethering have since relegated shunting to a secondary option due to its high complication and high recurrence rates (up to 92% at 3 years), inability to address the underlying cause of syringomyelia, and the need for further surgical procedures and frequent follow-up visits [7,25]. Patients treated with shunting have a seven times higher risk of developing recurrences than those who underwent arachnolysis [22]. Therefore, shunt operations are now considered a last resort when other treatments fail and the condition threatens neurological function. The primary indications for shunt placement include idiopathic syringomyelia and the failure of different therapies, with syringo-subarachnoid and syringo-peritoneal shunts being the most common procedures [7]. 

Syrinx shunting initially shows promising radiographic and clinical outcomes. Short-term follow-ups indicate a high rate of syrinx improvement (90.2%) with only 7.3% remaining unchanged, and 2.4% worsening. However, these positive outcomes diminish significantly over extended follow-up periods. With an average follow-up of approximately 31 months, the rate of syrinx improvement drops to 56%, with 39.2% remaining unchanged, and 4.8% worsening. This decline over time suggests that while shunting may offer effective short-term relief, its long-term efficacy is less reliable. While some reports show moderate success, particularly in spina bifida patients, long-term follow-up indicates a 50% failure rate over ten years if the primary cause is not addressed. Critics argue that short-term positive outcomes often diminish over time, with CSF diversion failing to prevent symptomatic recurrence [7]. This argument has been supported by findings of a study where the initial MRI follow-ups at three months showed 90% improvement in syrinx outcomes, but this decreased to 56% over extended follow-up periods (average 31 ± 28 months). Likewise, the syrinx’s aggravation also doubled with follow-up prolongation [22].

The primary objective of surgery for syringomyelia is to restore normal CSF flow dynamics, with the surgical approach tailored to the underlying cause of the condition [7]. In cases of Chiari-I malformation, a craniocervical decompression combined with a duraplasty is performed to enhance cerebrospinal fluid (CSF) flow, yielding the best outcomes [37]. Conversely, tonsil resection and shunt placement are discouraged due to the high incidence of side effects associated with tonsil resection and the potential for shunts to worsen clinical symptoms and enlarge cavities [37,38].

For Chiari-II malformation, the procedure typically includes enlarging the foramen magnum and removing cervical laminae to relieve pressure, along with restoring fourth ventricle outflow as needed. In spinal canal stenosis, a laminectomy can effectively restore CSF dynamics. Syringomyelia resulting from tumors or arachnoid cysts is treated through resection or fenestration of the lesions. However, surgical intervention for arachnoid scarring is particularly challenging due to the difficulty in distinguishing normal anatomical structures, often limiting options for direct drainage of the syrinx. Three drainage methods are available: syringo-arachnoid, syringo-peritoneal, and syringo-pleural. Syringo-arachnoid drainage is effective for localized scarring but may face shunt obstruction, while syringo-peritoneal drainage is less practical due to patient positioning. Syringo-pleural drainage offers advantages by removing CSF from the compromised arachnoid space and is more accessible during surgery, utilizing negative intrapleural pressure to facilitate drainage [8].

**An Illustrative case of a syringo-pleural shunt with a reservoir valve implant:**

**Figure 4:**
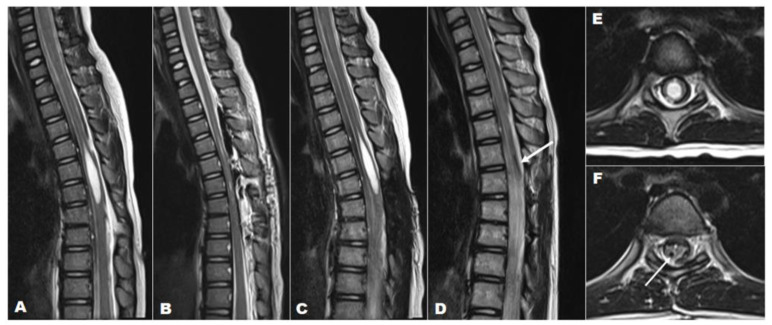
A 7-year-old girl with a large loculated syringomyelia, T4–T6, caused by a dermal sinus tract. Serial sagittal MRI images (T2-weighted) demonstrate (**A**) the preoperative syrinx (T4–T6) with TCS, (**B**) the complete syrinx resolution immediately post-untethering and simple drainage, (**C**) the syrinx recurrence at the previous location at 6 months post-untethering with almost the same size as the preoperative syrinx, and (**D**) the absence of the syrinx and the proximal catheter that was previously implanted (white arrow) at follow-up after 6 years post-shunt surgery. T2-weighted axial MRI indicating (**E**) the preoperative syrinx (SI > 0.8) and (**F**) the complete resolution of the syrinx and the proximal catheter (white arrow) at final follow-up 6 years after shunt surgery.

This illustrative case aims to demonstrate the application of a syringo-pleural shunt with a reservoir valve implant for the treatment of syringomyelia associated with TCS. A 7-year-old female was referred to our tertiary care hospital for a skin tag on her back and gait abnormalities. On history taking, it was noted that she had had an asymptomatic skin tag on her thoracic spine (Figure 4) since birth. However, it had become symptomatic, causing shock-like leg tingling sensations and episodic paraparesis when touched or pulled. A preoperative MRI revealed syringomyelia extending from T4 to T6 (SI > 0.8), which was associated with a dermal sinus tract through spina bifida at T7-8 (Figure 4). Initially, she underwent an osteoplastic laminotomy of T6–T8 with complete resection and reconstruction of the dermal sinus tract, along with a midline myelotomy and syrinx drainage at T6 (Figure 4). Following the surgery, an immediate postoperative MRI showed complete resolution of the syrinx; however, a recurrence of the syrinx, at the same size as preoperative, was noted in a follow-up MRI performed 7 months postoperatively. Finally, a syringo-pleural shunt using a reservoir valve was performed for the recurrent large syrinx. As a result, the patient exhibited both clinical and radiographic improvement and remained well during a follow-up period of 6 years.

This illustrative case demonstrates the application of a syringo-pleural shunt with a reservoir valve implant for the treatment of syringomyelia associated with TCS, particularly focusing on the limitations of direct drainage in managing a loculated large thoracic syrinx. Procedures like a terminal ventriculostomy or myelotomy and syringotomy are effective for treating terminal syringomyelia, especially when the syrinx extends to the conus. However, these direct drainage techniques have limitations in cases of loculated thoracic or cervical syrinxes, where shunt surgery may be a more viable alternative. The effectiveness of the syringotomy incision in terminal syringomyelia is likely due to its placement in a gravity-dependent part of the spine, which allows for direct drainage. The case highlights the use of shunt surgery in scenarios involving large lesions with a syrinx index greater than 0.5 and recurrent lesions. Additionally, the necessity and effectiveness of incorporating a reservoir valve are discussed to emphasize its role in optimizing clinical outcomes, particularly for appropriate drainage control. 

Syrinx shunting has been a common intervention for treating symptomatic syringomyelia, employing various techniques such as syringo-subarachnoid, syringo-peritoneal, and syringo-pleural shunts, which demonstrate differing rates of clinical improvement. While initial outcomes appear promising, with high rates of syrinx improvement in the short-term, the long-term effectiveness of shunting is significantly diminished as follow-up studies reveal a notable decline in improvement rates, with many patients experiencing clinical and radiological deterioration over time. The high recurrence rates and complications associated with shunting have prompted a reevaluation of its role in treatment, positioning it as a last resort when other therapies have failed and neurological function is at risk. The primary goal of surgical intervention should focus on restoring normal cerebrospinal fluid dynamics tailored to the specific etiology of syringomyelia, emphasizing the importance of addressing the root causes rather than relying solely on shunting. As advancements in surgical techniques continue to evolve, shunting is increasingly viewed as a secondary option, particularly indicated for loculated thoracic syringomyelia due to TCS with large cysts, especially when progressive symptoms are present and other treatments are inadequate or unfeasible. Further research and refinement of surgical techniques will be essential to improve patient outcomes and minimize the risks associated with syrinx shunting.

## 5. Conclusions

Surgical cord untethering effectively addresses the underlying causes of syrinx formation in many cases. However, larger syrinxes (index > 0.5–0.6) may necessitate additional interventions, such as a direct syringotomy/myelotomy or terminal ventriculostomy, to achieve optimal outcomes. 

As surgical techniques continue to advance, the emphasis has increasingly shifted towards addressing the root causes of syringomyelia for more sustainable results. Nonetheless, syrinx shunting remains a valuable last resort, particularly in cases where other treatments are inadequate or unfeasible, and when neurological function is at significant risk. Shunting is crucial for managing specific cases, such as large, loculated syrinxes, where direct drainage or other interventions have failed. Long-term management and careful monitoring are essential to address potential complications and ensure the best possible patient outcomes, with individualized treatment plans tailored to each patient’s unique clinical scenario.

## 6. Limitations of the Study

The study’s limitations stem from the scarcity of data on optimizing surgical approaches for syringomyelia associated with tethered cord syndrome, primarily relying on retrospective studies that introduce selection bias and variability in data collection. While the review provides valuable insights, it emphasizes the need for cautious interpretation of findings and further research in this area.

## Figures and Tables

**Table 1:** Summary of the literature on practicing surgical cord untethering alone for treating syringomyelia associated with TCS.

No. (Year)	Patients [M/F]	Etiologies of TCS (Case)	C/M of TCS (Case)	Preop. Syrinx Dimension and SI	Postop. Radiographic Result of the Syrinx	Outcome of the Syrinx (Case)	Overall Surgical Outcomes	Summary
2022 **[23]**	54 [23/31]	TF (40) 74% Sc (35) 64.8% DTM (31) 57.4% * SM (42) 78%	G1 (Ped, 34) u/b Dys (22) 64.5% mN/D (19) 55/8% Pain (16) 47% G2 (Adult, 20) Pain (15) 75% b Dys (6) 30%	G1: SI 0.21 ± 0.15 D 2.7 ± 1.9 × 2.8 ± 1.0 mm G2: SI 0.12 ± 0.17 D 2.8 ± 1.3 × 2.6 ± 1.6 mm Level T6–T12 (9) 22% T6–L5 (17) 40% L1–S1 (15) 36%	G1: SI 0.14 ± 0.15 D 2.1 ± 2.0 × 2.1 ± 1.7 mm G2: SI 0.05 ± 0.04 D 0.85 ± 0.7 × 0.8 ± 0.7 mm * Improved SI (*p* < 0.05)	G1 (27) 63% CR (5) 18.6% Improved (12) 44.4% U (10) 37% G2 (8) 87.5% CR (3) 37.5% Improved (4) 50% U (1) 12.5%	G1: 88% improved m/s N/D 70/70% u/b Dys 54.5% G2: 93% improved m/sN/D 86/84% u/b Dys 54.5%	- TCS is more severe when accompanied by terminal SM and/or other congenital anomalies, reducing the chances of postoperative recovery. - Close monitoring by a multidisciplinary team is crucial for the early detection of clinical or radiological changes.
2019 **[2]**	25 [17/8]	LMMC (8) 32% TF (7) 28% LP (5) 20% MCC (2) 8% MC (2) 8% DTM (1) 4%	Sc (11) 44% u/b Dys (8) 32% Pain (8) 32% mN/D (4) 16% sN/D (2) 8%	Lt 4.81 ± 4.35 mm WD 5.19 ± 2.55 mm Extent C4–T7 (cranial) C6–S2 (caudal)	Lt: 0.86 ± 4.36 mm (Incr.) W: 0.72 ± 2.94 mm (Dec.)	Improved (9) 36% U (12) 48% W (4) 16%	Improved: m/sN/D 4% u/b Dys 12% Pain 8% Sc w/fusion 16% U/W: m/sN/D 16% Pain 20% Sc w/o fusion 8/20%	- Surgery for TCS sometimes improved syrinxes, but not consistently, and did not always correlate with changes in Sx. - Surgical decisions should prioritize clinical symptoms over the mere presence of a syrinx.
2013 **[21]**	16 [8/8]	OSD (16) 100% TF (2) 12.5%	u Dys (12) 75% Pain (8) 50% Sc (3) 19% Gait ds (2) 12.5% B Dys (1) 6.2%	D: 3.7 mm (ranged 2–6.5 mm) Extent >T5 (6) 37.5% <T5 (10) 62.5% conus (10) 62.5%	D: 3.6 mm (overall) ➢ 3.4 mm in 25% ➢ 3.8 mm in 75%	Improved **: (4) 25% U ^+^ (12) 75%—no gait improvement, except 1 ** syrinx < T5 (4) ^+^ no improvement (6) in a cervical syrinx (*p* = 0.23).	Improved: u/b Dys 100% Pain 87.5% Sc 67% Gait ds 100% W: Sc 33%	- Some TCS patients showed significant clinical improvement, though only a quarter experienced radiographic syrinx improvement or resolution. - A syrinx and occult TCS may be incidental or even separate Sx of an underlying developmental process.
2012 **[20]**	33 [16/17]	LS LP (24) 73% TF (6) 18% LMMC (3) 9%	Asx (18) 55% mN/D (5) 15% Foot def (6) 18% sN/D (1) 3% u Dys (7) 21% b Dys (7) 21%	G1: SI < 0.6 (20) 60% G2: SI ≥ 0.6 (13) 39% SI 0.4–0.7 (24) 72% SI > 0.7 (9) 27% Lt: 3.4 ± 3.0 (vert. level) Location: TL (16) 48% LS (17) 52%	No statistical postop changes in two groups (SI of <60 vs. >60)	CR (8) 25% Improved (10) 31% U ^++^ (9) 28% W (1) 3%	GI: SI < 0.6 (n = 20) Improved/U 90% W 10% GII: SI ≥ 0.6 (n = 13) improved/U 61% W 23%	- Untethering alone, in TCS and SM associated with occult SD is an effective management strategy and has favorable postop. including both clinical and radiological outcomes. - Postop. Asx. syrinx enlargement may resolve spontaneously.

Abbreviation: Asx = asymptomatic, C = cervical, D = diameter, DTM = diastematomyelia, Foot def = foot deformity, Gait ds = Gait disorder, L = lumbar, LP = lipoma, LMMC = lipomyelomeningocele, LS = lumbosacral, Lt = length, MC = meningocele, MC = meningocele, mN/D = motor neurological deficit, OSD = occult spinal dysraphism, Postop = postoperative, Sc = scoliosis, SD = spinal dysraphism, SI = syrinx index, SM = syringomyelia, sN/D = sensory neurological deficit, Sx = symptoms, T = thoracic, TCS = tethered cord syndrome, TF = tick filum, TL = thoracolumbar, U = unchanged, u Dys = urinary dysfunction, b Dys = bowel dysfunction, W = Worse, and WD = width. G1 = group 1 (pediatric patients, aged < 18 years) and G2 = group 2 (adults, aged > 18 years). GI = first group and GII = second group. * = Syringomyelia accompanying pathology of TCS. ^++^ Initially aggravated, and stable after 6 mo (4).

**Table 2:** Summary of the literature on practicing direct syrinx drainage and cord untethering for treating syringomyelia associated with TCS.

No. (Year)	Patients [M/F]	Etiologies of TCS (Case)	C/M of TCS (Case)	Preop. Syrinx Dimension and SI	Postop. Radiographic Result of the Syrinx	Outcome of the Syrinx (Case)	Overall Surgical Outcomes	Summary
2023 **[24]**	28 [12/16]	TCS (26) 93% DTM (1) 3.6%	m N/D (24) 85.7% s N/D (19) 67.9% u/b Dys (12) 43% constip. (7) 25% Amyotr. (5) 17.9% foot df (3) 10.7% Asx (1) 3.8%	SI.: 0.49 ± 0.16 Trans. 4.5 ± 1.1 mm Centric (25) 89.3% Eccentric (3) 14.3% Level >10 seg (13) 46.4% 5–10 seg (11) 39.3% <5 seg (4) 14.3%	SI: 0.06 ± 0.08 (improved, *p* < 0.001) Resolved (21) 75% Improved (7) 25%	SM shrunk—50% None relapsed, 3 yrs Surgery (28): Untethering + TV	I: m/s N/D 91/79% uDys 33% constip. 14% U: m/s N/D 8.3/21% uDys 67% constip. 86% Foot def 100% Asx 100%	- TV is safe, convenient, and consistently effective for terminal SM, particularly for syrinxes extending to the filum terminale.
2007 **[19]**	34 [17/17] G1 (24) G2 (10)	G1: TCS (24) FM (19) 79% LP (9) 37.5% MC (2) 8.3% MMC (2) 20% G2: TCS + TS(10) FM (7) 70% LP (3) 30% MMC (2) 20% DTM (1) 10%	G1: TCS (24) Asx: (9) 37.5% m/s N/D (4/1) 16.7/4.2% u/b Dys (5) 21% Sc (1) 4.2% Pain (4) 16.7% G2: TCS + TS (10) Asx (5) 50% m/s N/D(1) 10% u/b Dys (3) 30% Sc (1) 10% Pain (4) 40%	No SI value Lt: 4.6 seg. (mean, 2~10) Level: midT (2) 20% TL (3) 30% LS (5) 50%	Postop MR (3/10) in G2 Improved (2/3) 66% U (1/3) 33%	Reduced size (2) at 4 and 7 mos MRI U (1): on 2 yr MR Surgery: All Untethering (34) Direct drainage (1) * * G2: >1.5 cm SM	G1: Improved 60% Asx 20% U 13.3% W 6.6% G2: Improved 80% Asx 20%	- Untethering surgery alone effectively improves clinical Sx without the need for additional syrinx drainage. - There is no evidence that combining syrinx drainage with untethering surgery enhances clinical outcomes or affects postop results.
2000 **[18]**	30 [11/19] G1 (16) G2 (14) - tSM	TF (12) 40% DTM (13) 43.3% Prev OP (4) 14% DTM + LP(1) 3.3%	m/s N/D (23/26) 76.6/86.6% u/b Dys (20/11) 70/36.6% Sc (15) 50% Pain (10) 33.3% Gait dys (13) 43.3%	G1: SI 0.40 ± 0.20 G2: SI 0.45 ± 0.16 Trans./Sag. (mm) G1: 3.0 ± 1.7/13.3 ± 1.8 G2: 3.0 ± 1.4/4.0 ± 1.2 Centric (4/4) 29/29% Eccentric (11/10) 75/71% Lt: G1: 4.2 ± 1.7 seg G2: 4.3 ± 1.6 seg	G1: SI 0.31 ± 0.13 (*p* = 0.13) G2: SI 0.18 ± 0.11 (*p* = 0.001) G1: improved (7) 42.8% U (9) 56.2% W * (1) 6.2% * no FU data G2: CR (2) 14.2% improved (9) 64.2% U (3) 21.4%	* Higher sN/D and u/bDys improvement in cases of reduced SM over no SM change (*p* = 0.019) Surgery: G1: Untethering alone (16) G2: with TV (8) or myelotomy (M, 6)	G1: I: m/s N/D 50/50% u/b Dys 30% U: m/s N/D 43/50% u/b Dys 70% W: m N/D 1% G2: I: m/s N/D 78/92% u/b Dys 70% U: m/s N/D 11/8% u/bDys 27% W: m/sph N/D 11/7%	- tSM should be seen as a contributing factor in TCS. - Better outcomes occur when both the syrinx is decompressed and the spinal cord is untethered. - Surgical Tx should consider intramedullary distention, dysplastic cord anatomy, and Sx progression.
1999 **[17]**	32 [10/22] G1 (19) G2 (13)	132 TCS TF (12) 37% LP (25) 78% DTM (31) 96% rLMMC (16) 50% LMMC + LP (16) 50%	m/sN/D (22/20) 68.8/62.5% u/bDy (14/7) 43.8/21.9% Sc (15) 46.9% Blts (11) 37.5% Pain (4) 12.5%	SI: 0.42 ± 0.2 WD: 3.6 ± 2.1 mm Centric (9) 28% Eccentric (23) 72% Level T8–T12 38% T8–L4 28% L1–S1 34%	G1: improved (5) 26% U (14) 73% G2: CR (4) 30.7% improved (5) 38.4% U (4) 30.7%	Improved clinical outcomes in G2 highlight the importance of identifying and addressing this pathological condition Surgery: G1: Untethering alone (19) G2: untethering with ① TV (7) ② M (5) ③ Shunt (1)	Overall Improved in - m/s N/D 75/71.4% - b Dys 60% Improved G1 vs. G2 - mN/D: * 7% vs. * 22% - sN/D * 68% vs. * 75% - Sph Dys 57% vs. 63% (* marked and slight) Unchanged G1 vs. G2 - mN/D: 33% vs. 21% - sN/D 32% vs. 25% - Sph Dys 43% vs. 37%	- Patients with a syrinx have higher rates of neurological Sx, including Sph Dys and progressive Sc. - Large paracentral cavitations in tSM are more common and significant than small central dilations. - Radiologically significant tSM worsens TCS by exacerbating neurological deficits.

Abbreviation: Amyotr = amyotrophy, Blts = bilateral long tract signs, constip = constipation, DTM = diastematomyelia, FM = fatty filum, FU = follow-up, G1 = first group, G2 = second group, L = lumbar, Lt = length, LP = lipoma, LS = lumbosacral, LMMC = lipomyelomeningocele, M = myelotomy, MC = meningocele, MMC = myelomeningocele, m N/D = motor neurological deficit, Postop = postoperative, rLMMC = repaired lipomyelomeningocele sites, Sc = scoliosis, SI = syrinx index, SM = syringomyelia, s N/D = sensory neurological deficit, Sph Dys = sphincter dysfunction, Sx = symptoms, TF = tick filum, TL = thoracolumbar, tSM = terminal syringomyelia, TCS = tethered cord syndrome, TV = terminal ventriculostomy, U = unchanged, u/b Dys = urinary/bowel dysfunction, u Dys = urinary dysfunction, b Dys = bowel dysfunction, Tx = treatment, W = Worse, and WD = width, * No further follow-up data is available.

**Table 3:** Summary of the literature on practicing syrinx shunting for treating syringomyelia associated with TCS.

No. (yr)	Patients [M/F]	Etiologies of TCS (Case)	C/M of TCS (Case)	Preop. Syrinx Dimension and SI	Postop. Radiographic Result of the Syrinx	Outcome of the Syrinx (Case)	Overall Surgical Outcomes	Summary
2018 **[22]**	SM (41)	* Etiology of SM unknown (15) 37% Known (26) 63% - 13 trauma - 5 CM - 4 arachnoiditis - 3 TCS (7.3%) - 1 band	Pain 6 15% sN/D (17) 42.5% mN/D 11) 27% MP (2)5% Spas (4) 10% uDys (1) 2.5%	D: >2 mm Level: C (11) 27% CT (5) 12% CL (2) 5% T (18) 44%	Initial FU (3 mo) Improved: (37) 90.2% U: (3) 7.3% W: (1) 2.4% Final FU (31 ± 28 mo) Improved: (23) 56% U: (16) 39.2% W: (2) 4.8%	Reduced size (37): outcome varied over time Surgery: SS shunt * subset—expansile duraplasty	TCS (3) I (3) 100% Others I (29/32) U (8) 19.5% W (1) (trauma)	- The pathophysiology of each SM should guide treatment. - SS shunting is safe and effective for idiopathic SM or unknown causes, or when first-line treatment is insufficient or not feasible. - All improved or stabilized Sx except one worsened. - Reoperation Rate of 7% at average FU of 108 mos.
1998 **[16]**	SM (32) - Asx (18) - Sx (14) SD (142)	MMC (14) 100% Asx of SM (18) - Group a: I/II (7/2) - Asx of SD I/II (4/5) Group b: II/III (5/9)	mN/D (10) 71.4% Sc (8) 57.1% Pain (5) 35.7% Pyr. Sxl (4)28.6% sN/D (3) 21.4% Neck Sx (2) 21.4% uDys (3) 21.4%	SI: Severe (9) 64.3% Moderate (5) 35.7% Extent: Holocord: (6) 42.9% CT (3) 21.4% T (3) 21.4% TL (2) 14.2%	CR (4) 28.5% * % of SM changes a. <50% decr.(2) 14.2% b. >50% decr.(8) 57.1% * A correlation btw the Sx and syrinx	Shunt (7): SP shunt (5), SS shunt (2) Indirect Tx (8): SOC (5), VP shunt (2), untethering (1)	mN/D (10): CR/I/St: (6/2/2) Sc (8): I/St: (3/5) Pain (5): CR: (5) Pyr. sg (4): CR: (4) sN/D (3): I: (3) Neck stiff (3): CR/I: (2/1) uDys (3): I: (3)	- Improvement in clinical status correlates with a reduction in SM size. - All improved, but one underwent SOC followed by SS shunt - The radiographic appearance of SM indicates clinical severity. - Progressive Sc signals SM deterioration. - Surgical treatment of SM has very good outcomes.
1997 **[15]**	SM (15) [4/11] SD (43)	OSD (34) 80% OSD + SM (8) 23.5% MMC (9) 20% MMC + SM (9)100%, FU only 7	OSD (34) mN/D (3) 38% u/bDys (7) 87.5% MMC (9) mN/D (7) 100% sN/D (7) 100% u/b Dys (7) 100% Nyst (3) 43%	OSD: SI ≤ 0.7 (7) 87.5% large SI ≥ 0.7 (1) 12.5% Level: LS (8) 100% MMC large SI ≥ 0.7 (3) 43% Level: CT (3) 42% CL (4) 57%	SS shunt (6): - Collapse of SM (6) MMC/hydrocephalus: - VP shunt All: No Cx, No Neurological deterioration.	OSD: untethering and - SS shunt (2), - syringostomy (2) OSD: Untethering alone - Improved (3/8) 38% * Other SS shunt (6): U MMC: - SS shunt (2) - Collapse of SM (6)	OSD SM (8) No N/D (7) mN/D (1) MMC CM (7): Plasty + VP shunt (7) * SS hunt for Large SM (2) Improved (1) No mN/D change (6)	- Large symptomatic syrinxes (SI ≥ 0.7) with OSD should be surgically treated. - SS shunting is effective for both closed and OSD-associated SM. - Foramen magnum decompression is indicated for SM with MMC. - Large symptomatic SM in MMC was successfully treated with SS shunting.
1994 **[14]**	tSM(27) 33.2% 138 OSD (143) 57:86	OSD c MRI (90) DST (6) 5.4% LMMC (41) 37% TC (9) 8.1% DTM (26) 23.4% MC (11) 9.9% TC_a (9) 8.1%	OSD with tSM (27) N/D (11) 40.7% u/bDys (13) 48.1% foot-df (13) 48.1% Sc (16) 59.3% Pain (9) 33.3% Asx (4) 15%	tSM (27) A. Large (17): SI > 0.5 and L2 cm - SM ends < T6 (16) - to L (9)/T (7)/S(4) - 1 holocord SM B. Small (10): < 0.5 - at Conus (5) - <T-6 (5)	Large SM-Shunt (12) CR (5): A(2), B(3) I (5): A(2), B(3) U (1): A (1) A: shunt (6) B: shunt + OSD (6)	Shunt (12 for Large SM): CR/I (10) 91% - All 5 Pain: CR (5) - 1/3 (4): improved, U (1) d/t unusual op site scar	Shunt (12): Pain (5): I (5) 100% N/D: improved (4), U (7): No Sx worsen	- Shunting is necessary and effective for SM with a large cyst, especially with progressive Sx. - TS is important and should be considered in OSD patients; treated surgically when clinically or radiographically significant.

Abbreviation: Asx = asymptomatic, C = cervical, CL = cervicolumbar, CM = Chiari malformation, CR = complete resolution, CT = cervicothoracic, DST = dermal sinus tract, DTM = diastematomyelia, FU = follow-up, I = improved, L = lumbar, LS = lumbosacral, LMMC = lipomyelomeningocele, MC = meningocele, MMC = myelomeningocele, mN/D = motor neurological deficit, MP = myelopathy, N/D = neurological deficit, Postop = postoperative, Sc = scoliosis, SD = spinal dysraphism, SM = syringomyelia, sN/D = sensory neurological deficit, SOC = suboccipital craniectomy, spas = spasticity, SS = syringo-subarachnoid, Sx = symptoms, T = thoracic, TC = tethered cord, TC_a = tethered cord with anorectal anomaly, TCS = tethered cord syndrome, TL = thoracolumbar, tSM = terminal syringomyelia, U = unchanged, foot-df = foot deformity, u Dys = urinary dysfunction, u/b Dys = urinary/bowel dysfunction, and W = Worse. I, II, and III indicate the ratio of hydromyelia diameter to that of the total spinal cord corresponding to 0.25–0.33, 0.33–0.5, and >0.5, respectively. * The etiologies refer to syringomyelia.
